# A Simple and Sensitive Liquid Chromatography with Tandem Mass Spectrometric Method for the Simultaneous Determination of Anthraquinone Glycosides and Their Aglycones in Rat Plasma: Application to a Pharmacokinetic Study of *Rumex acetosa* Extract

**DOI:** 10.3390/pharmaceutics10030100

**Published:** 2018-07-20

**Authors:** Hossain Mohammad Arif Ullah, Junhyeong Kim, Naveed Ur Rehman, Hye-Jin Kim, Mi-Jeong Ahn, Hye Jin Chung

**Affiliations:** College of Pharmacy and Research Institute of Pharmaceutical Sciences, Gyeongsang National University, Jinju 52828, Korea; arifpha@ymail.com (H.M.A.U.); jhk6914@naver.com (J.K.); naveed.rehman50@gmail.com (N.U.R.); black200203@gmail.com (H.-J.K.)

**Keywords:** anthraquinone, glycoside, aglycone, LC-MS/MS, plasma, protein precipitation

## Abstract

*Rumex acetosa* (*R. acetosa*) has been used in folk remedies for gastrointestinal disorders and cutaneous diseases. *Rumex* species, in particular, contain abundant anthraquinones. Anthraquinone glycosides and aglycones show different bioactive effects. However, information on the pharmacokinetics of anthraquinone glycosides is limited, and methods to quantify anthraquinone glycosides in plasma are rarely available. A simple and sensitive liquid chromatography-tandem mass spectrometric bioanalytical method for the simultaneous determination of both anthraquinone glycosides and their aglycones, including emodin, emodin-8-*O*-β-d-glucoside, chrysophanol, chrysophanol-8-*O*-β-d-glucoside, physcion, and physcion-8-*O*-β-d-glucoside , in a low volume of rat plasma (20 µL) was established. A simple and rapid sample preparation was employed using methanol as a precipitating agent with appropriate sensitivity. Chromatographic separation was performed on HPLC by using a biphenyl column with a gradient elution using 2 mM ammonium formate (pH 6) in water and 2 mM ammonium formate (pH 6) in methanol within a run time of 13 min. The anthraquinones were detected on triple-quadrupole mass spectrometer in negative ionization mode using multiple-reaction monitoring. The method was validated in terms of selectivity, linearity, accuracy, precision, recovery, and stability. The values of the lower limit of quantitation of anthraquinones were 1–20 ng/mL. The intra-batch and inter-batch accuracies were 96.7–111.9% and the precision was within the acceptable limits. The method was applied to a pharmacokinetic study after oral administration of *R. acetosa* 70% ethanol extract to rats at a dose of 2 g/kg.

## 1. Introduction

*Rumex acetosa* L. (*R. acetosa*), belonging to the Polygonaceae family, is a perennial herb that is listed in the Korean Food Code (Korea Food and Drug Administration) as a food material and has been used in folk remedies for gastrointestinal disorders and cutaneous diseases [1]. Extracts of *R. acetosa* have been reported to have various biological activities, including anti-ulcerogenic, anti-inflammatory, anti-proliferative, and anti-viral effects [2,3,4]. They contain a number of bioactive compounds, including anthraquinones, flavonoids, and polysaccharides [5]. In particular, *Rumex* species contain abundant anthraquinones, including emodin, chrysophanol, and physcion in all parts of the plant, in free and glycoside forms [6]. A difference in anthraquinone physiological activity between these forms has been described [7]. Previous studies reported a number of quantitative methods of anthraquinones in plasma [8,9,10]. However, we found that most of these studies focused on determining aglycones (free anthraquinones). Methods to quantify anthraquinone glycosides in plasma are rarely available.

As interest in natural drugs has increased in the pharmaceutical industry, research is underway to develop potential applications of *R. acetosa*, which has already proven its efficacy. Therefore, a simple and sensitive analytical method to examine bioactive anthraquinones in biological samples is needed to evaluate the potential of new treatments. 

The aim of this study is to establish a simple, rapid, and sensitive liquid chromatography-tandem mass spectrometry (LC-MS/MS) method to simultaneously quantify emodin (E), emodin-8-*O*-β-d-glucoside (EG), chrysophanol (C), chrysophanol-8-*O*-β-d-glucoside (CG), physcion (P), and physcion-8-*O*-β-d-glucoside (PG) in rat plasma within one chromatographic run. The method was applied to determine pharmacokinetic parameters after oral administration of *R. acetosa* 70% ethanol extract in rat. The results of this study might be helpful in the development of a new type of medicine using *R. acetosa*. 

## 2. Materials and Methods

### 2.1. Materials

**Figure 1:**
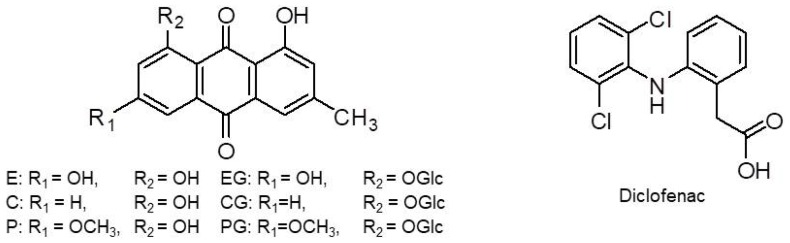
The chemical structures of six anthraquinones and diclofenac (internal standard). E, emodin; EG, emodin-8-*O*-β-d-glucoside; C, chrysophanol; CG, chrysophanol-8-*O*-β-d-glucoside; P, physcion; PG, physcion-8-*O*-β-d-glucoside.

The plant of *R. acetosa* L. (Polygonaceae) was collected from the Sancheong province of Korea in April 2014 and identified by Mi-Jeong Ahn of the College of Pharmacy, Gyeongsang National University (Jinju, Korea). The voucher specimen (APG-1403) was deposited in the Herbarium of the College of Pharmacy, Gyeongsang National University. The standards of the six anthraquinones (E, EG, C, CG, P, and PG) were isolated from the whole part of *R. acetosa* and their structures (Figure 1) were elucidated using spectroscopy such as MS and nuclear magnetic resonance spectroscopy (data not shown) [11]. The purity of anthraquinone compounds isolated from *R. acetosa* was confirmed to be more than 95% by NMR and HPLC-UV. Diclofenac used as an internal standard (IS) was purchased from Sigma Aldrich (St. Louis, MO, USA). HPLC-grade acetonitrile, methanol, and water were products of Fisher Scientific Korea Ltd. (Seoul, Korea). All reagents were analytical grade.

### 2.2. Chromatographic Condition

The analysis was performed on an Agilent 1260 series (Agilent Technologies, Waldbronn, Germany) HPLC system. Chromatographic separation of the samples was carried out on a Kinetex Biphenyl column (100 × 3.0 mm, 2.6 μm, 110 Å, Phenomenex, Torrance, CA, USA). The mobile phase consisted of 2 mM ammonium formate (pH 6) in water (A) and 2 mM ammonium formate (pH 6) in methanol (B). The gradient program was used at a flow rate of 0.3 mL/min while maintaining the column temperature at 40 °C. The mobile phase initial composition of 25% B was maintained for 2 min. It was then increased linearly from 25% to 95% B for 0.5 min and held for 7 min. The gradient was then changed back to the initial condition for 0.5 min and kept at the initial condition for 3 min. The total analysis time was 13 min for each sample. The injection volume was 15 μL. 

### 2.3. Mass Spectrometric Condition

**Figure 2:**
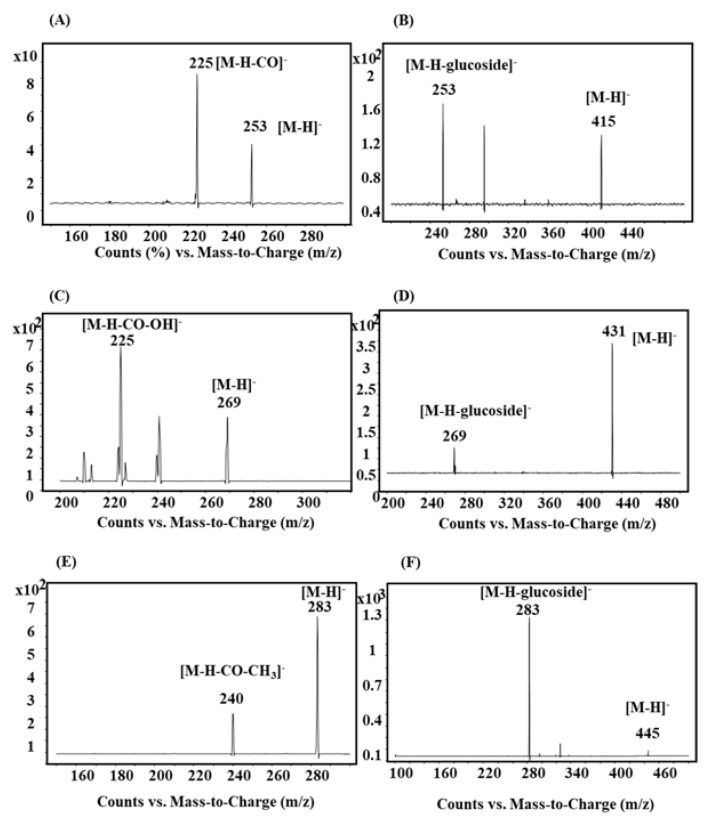
MS/MS scan spectra of six anthraquinones. (**A**) chrysophanol; (**B**) chrysophanol-8-*O*-β-d-glucoside; (**C**) emodin; (**D**) emodin-8-*O*-β-d-glucoside; (**E**) physcion; (**F**) physcion-8-*O*-β-d-glucoside.

The mass spectrometric detection was performed on an Agilent 6460 triple-quadruple mass spectrometer (Agilent Technologies, Singapore) with an electrospray ionization source. It was operated in the negative ion detection mode because of its higher sensitivity than that in the positive ionization mode on multiple reaction monitoring (MRM). The data were acquired and processed using Mass Hunter Workstation B.06.00 software (Agilent Technologies, Singapore). The mass spectrometric parameters of each compound are summarized in Table 1 [12]. The MS spectra of the six anthraquinones are shown in Figure 2. The source parameters were also optimized as follows: a drying gas flow and temperature at 6 L/min and 350 °C were used, respectively; the sheath gas flow and temperature were maintained at 12 L/min and 350 °C, respectively; the nebulizing gas (N_2_) pressure was set at 25 psi; and the capillary and nozzle voltages were set at 3500 V and 500 V, respectively.

### 2.4. Preparation of R. acetosa Extract

The dried plant material (100 g) was ground and extracted with 70% ethanol. The extract was filtered using filter papers (Whatman No. 40) and concentrated through a rotary evaporator. The concentrate was lyophilized and stored at −80 °C. The exact amount was weighed and used as the samples for the animal studies. The contents of E, EG, C, CG, P, and PG in *R. acetosa* extract were 0.94 ± 0.15%, 1.29 ± 0.06%, 0.68 ± 0.09%, 0.77 ± 0.12%, 0.17 ± 0.02%, and 0.41 ± 0.05% (*w*/*w*), respectively. The values were expressed as mean ± standard deviation. 

### 2.5. Preparation of the Calibration Standard and Quality Control (QC) Samples

The primary stock solutions of E, EG, C, CG, P, PG, and IS were prepared in dimethyl sulfoxide at a concentration of 1 mg/mL and stored at −80 °C. The mixture stock solutions to obtain the standard solutions were serially diluted in methanol. The IS stock solution of 5 ng/mL was prepared in methanol. The calibration standards were prepared by spiking 10 μL of above standard solutions into 90 μL of blank rat plasma to yield concentration ranges of 1–300 ng/mL for E, 20–300 ng/mL for P and C, 1–150 ng/mL for EG, 10–150 ng/mL for CG and PG. Twenty microliters of aliquots were prepared and stored at −80 °C until analysis.

The QC samples were prepared in the same way as the calibration samples for E, EG, C, CG, P, and PG in rat plasma at low, middle, and high concentrations. All the solutions were kept at −80 °C.

### 2.6. Sample Preparation

To 20 μL aliquot of the rat plasma samples, 60 μL of 5 ng/mL IS in methanol was added. The mixture was vortexed for 30 s and kept at 4 °C for 30 min. The mixture was centrifuged at 10,000× *g* for 10 min. The supernatant was transferred to an HPLC vial, and 15 μL of the processed sample was injected onto the LC-MS/MS system.

### 2.7. Method Validation

The method validation was performed according to the United States Food and Drug Administration’s guidance on bioanalytical method validation [13].

#### 2.7.1. Selectivity

The selectivity study was performed by comparing the chromatograms of the six different rat plasma samples to investigate the interference near the retention time of the analytes and the IS. 

#### 2.7.2. Calibration Curves and Sensitivity

The linearity of each calibration curve was determined by plotting the peak area ratio of the analyte to IS versus the plasma concentrations. The least-square method was used to achieve a linear regression equation. Sensitivity was defined by calculating the lower limit of detection and the lower limit of quantification (LLOQ) based on a signal-to-noise ratio of greater than 3 and 10, respectively. Besides signal-to-noise ratio, LLOQ values with acceptable precision and accuracy values were chosen. The criteria of precision and accuracy at LLOQ are within 20% relative standard deviation (RSD) for precision and between 80–120% for accuracy.

#### 2.7.3. Precision and Accuracy

Precision and accuracy were investigated by analyzing six replicates of four QC levels on the same batch (intra-batch) and five different batches (inter-batch) of four QC levels (LLOQ, low QC, middle QC, and high QC). The intra- and inter-batch precision was expressed by RSD (%), and accuracy was evaluated by expressing it as a percentage of the theoretical value (the mean calculated concentration/nominal concentration) × 100%. The acceptance criteria are within 15% RSD except 20% at LLOQ for precision and ±15% of nominal concentrations except ±20% at LLOQ for accuracy.

#### 2.7.4. Extraction Recovery and Matrix Effect

The extraction recovery was evaluated by comparing the peak area of the extracted sample with that of the post-extracted sample at three replicates of three QC levels. The matrix effect of the analytes was investigated by comparing the peak area of the post-extracted sample with the peak area obtained by the corresponding standard solutions in pH 7.4 buffer at three QC levels. Matrix effects were determined using the equation below.

$$\left(\frac{\text{peak area of the analytes for the sample spiked with the target compounds after extraction}}{\text{peak area of the analytes for the standard solutions}}\right)\times 100\%$$

#### 2.7.5. Stability

The stability of the analytes in rat plasma was evaluated by analyzing triplicates of three QC levels at room temperature for 4 h (short-term stability), −80 °C for one month (long-term stability), three freeze-thaw cycles from −80 °C to room temperature (freeze and thaw stability), and 4 °C for 24 h (processed sample stability). The stability of analytes in stock solution was also evaluated. The peak areas obtained from freshly prepared stock solutions were compared with stock solutions stored for 4 h at room temperature.

### 2.8. Pharmacokinetic Study

Male Sprague‒Dawley rats (8-week-old, weighing 250 ± 10 g) were obtained from Koatech (Pyeongtaek, Korea). They were housed and acclimated in the Animal Laboratory, Gyeongsang National University, under controlled temperature and humidity and regular 12 h light cycle, freely accessible to food and water for 7 days before the experiment. The rats were cannulated into the carotid artery and allowed to recover for one day. Before the pharmacokinetic study, all rats were fasted for 12 h with free access to water. *R. acetosa* extract suspended in a solution (ethanol:polysorbate 80:water = 1:2:7, *v*/*v*/*v*) was orally administered to the three rats at a dose of 2 g/kg. The calculated doses of compounds based on the contents in the extract were 18.8, 25.8, 13.6, 15.4, 3.4, and 8.2 mg/kg for E, EG, C, CG, P, and PG, respectively. Blood samples (100 µL) were collected via the cannulated carotid vessel at 0, 15, 30, 45 min, 1, 2, 3, 4, 6, 8, 12, and 24 h after oral administration. To collect plasma, the blood samples were immediately centrifuged at 10,000× *g* for 5 min. All plasma samples were stored at −80 °C until analysis. All experimental procedures of the animal study were approved (GNU-130618-R0038) by the Animal Care and Use Committee of Gyeongsang National University, Korea.

## 3. Results

### 3.1. Method Validation

#### 3.1.1. Specificity and Selectivity

**Figure 3:**
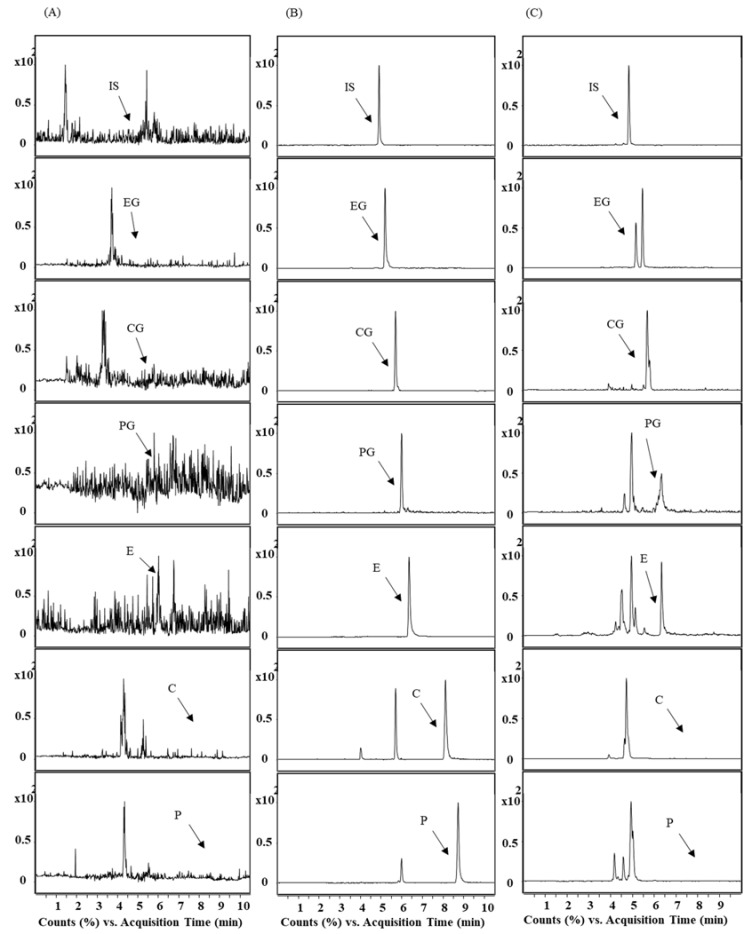
Representative MRM chromatograms of IS, EG, CG, PG, E, C, and P in rat plasma. (**A**) blank plasma; (**B**) blank plasma spiked with six anthraquinones (250 ng/mL for aglycones and 125 ng/mL for glycosides) and IS; (**C**) plasma sample obtained from rats 45 min after oral administration of *R. acetosa* extract (2 g/kg).

The representative MRM chromatograms of blank rat plasma and that spiked with six anthraquinones and IS are shown in Figure 3. No interference from endogenous substances near the retention time of the analytes or the IS was observed. 

#### 3.1.2. Linearity and Sensitivity

The calibration curves showed good linearity over their corresponding ranges for the analytes (R^2^ > 0.9934).

#### 3.1.3. Precision and Accuracy

The intra- and inter-batch precision and accuracies are presented in Table 2. The RSD values for the intra- and inter-batch were below 13.5%, except for EG at LLOQ (18.9%). The accuracies were between 85% and 115%. All results showed acceptable accuracy and precision.

#### 3.1.4. Extraction Recovery and Matrix Effect

The extraction recoveries and matrix effects of the anthraquinone compounds are shown in Table 3. The matrix effects were consistent among different concentrations for each compound. The recoveries ranged from 96.0% to 112.7% for the analytes at the QC levels. The matrix effects were constant for each analyte with different concentrations.

#### 3.1.5. Stability

The stability of stock solution was determined. As compared with fresh stock solutions, the mean concentration of analytes (*n* = 3) in stock solutions stored at room temperature for 4 h were 99.2, 94.8, 95.9, 102.4, 100.4, and 101.7% for P, E, C, PG, EG, and CG, respectively. There was no detectable degradation of compounds in dimethyl sulfoxide and methanol stored at room temperature for 3 months based on HPLC-UV chromatogram. The stability results of the analytes in rat plasma under different conditions are shown in Table 4. 

### 3.2. Pharmacokinetics Study

**Figure 4:**
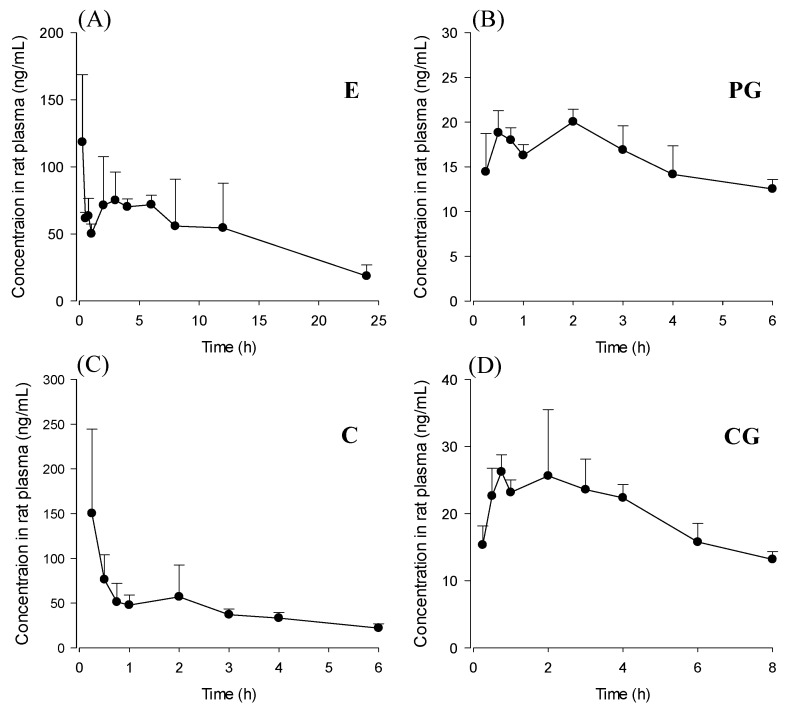
Mean plasma concentration–time profiles after oral (*n* = 3) administration of *R. acetosa* extract (2 g/kg) to SD male rats. Bars represent standard deviation. (**A**) emodin; (**B**) physcion-8-*O*-β-d-glucoside; (**C**) chrysophanol; (**D**) chrysophanol-8-*O*-β-d-glucoside.

The validated LC-MS/MS method was applied to the pharmacokinetic study after oral administration of *R. acetosa* extract at a dose of 2 g/kg to the rats. The concentrations of EG and P were not high enough to determine the pharmacokinetic parameters. The concentrations of C and PG were below the LLOQ from 6 h after administration of extract, CG and E could be detected until 8 and 24 h, respectively. The mean plasma concentration–time profiles of the analytes are presented in Figure 4. The major pharmacokinetic parameters of C, E, CG, and PG calculated by non-compartmental analysis are listed in Table 5. The data were expressed as mean ± standard deviation. The concentrations of emodin fluctuated and were insufficient to calculate the half-life of elimination (t_1/2_). Meanwhile, rapid absorption of aglycones was observed because of the higher lipophilic character with T_max_ of 0.25 (0.25–0.5) h and 0.25 (0.25–0.75) h, compared with that of glycosides.

## 4. Discussion

The objective of this study was to develop a bioanalytical method that simultaneously quantified the bioactive glycosides and aglycones of anthraquinones. The developed LC-MS/MS method could quantify six anthraquinones simultaneously in rat plasma in an accurate, reproducible, and simple way. Reported bioanalytical methods which simultaneously determine both aglycones and glycosides of anthraquinones are rarely available. In this study, simultaneous determination achieved by using biphenyl column. The column could prolong retention time of hydrophilic glycosides compare to C18 column at the same mobile phase composition. 

There was no interfering peak when the compound mixture was spiked to blank rat plasma. However, there were small peaks appeared near CG and PG peaks after oral administration of plant extract. It is suggested that those peaks came from the extract or the metabolites of components in the extract. It is known that emodin is extensively glucuronized after absorption [14] and the molecular weight of emodin glucuronide is same as PG. There is some possibility that emodin glucuronide could interfere PG. However, the MS/MS fragment pattern of emodin glucuronide is different from PG. Emodin glucuronide might cause little interference. We could quantify CG and PG by adjusting the baselines because the interfering peaks were small. 

A simple and rapid sample preparation was utilized on a low volume of rat plasma sample (20 µL) by using methanol as a precipitating agent with appropriate sensitivity compare to the reported methods [8,12,15]. The comparison with reported analytical methods for aglycones and glycosides of anthraquinones was shown in Table 6. 

The method was acceptably validated and used to perform a pharmacokinetic study of anthraquinones after oral administration of *R. acetosa* in rats. As shown in Figure 4, C, E, CG, and PG could be detected in every rat from the first sampling time, 15 min. All of the studied anthraquinones were absorbed rapidly from rat gastrointestinal tract. Median T_max_ value of C and E was 15 min (Table 5). This result was consistent with reported values [8,10]. Emodin could be detected for the longest time among four compounds even though the concentrations fluctuated. The fluctuated concentration was also reported in other pharmacokinetic studies of emodin in rats [9,15]. This was possibly due to enterohepatic circulation [16]. In some other works [17], emodin rapidly and extensively metabolized to form its glucuronide and the parent form was almost undetectable after administration of emodin even the doses were similar (40 mg/kg) to our study (18.8 mg/kg for E and 25.8 mg/kg for EG). Free emodin could be measured until 24 h after oral administration because of the low LLOQ level of emodin using the method developed in this work. 

Generally, plant glycosides have been considered to be hydrolyzed to aglycones by microflora in the gastrointestinal tract before absorption [18]. Glycosides have large molecular weights and low lipophilicity, so they might be difficult to be absorbed. However, recent studies show that emodin glycoside can be absorbed in an intact form after oral administration of plant extract [8]. The absorption of the glycosides of anthraquinones in an intact form was confirmed by studying in vivo absorption in rats in this study. Pharmacokinetics of anthraquinone aglycones and their glycosides after oral administration of *R. acetosa* was first evaluated. CG and PG were detected in rat plasma after oral administration of *R. acetosa* extract. Interestingly, the T_max_ of C and that of CG were different after oral administration of the extract. It might be due to different lipophilicity. Glycosides and aglycones have been proposed to have different degrees of absorption and metabolic patterns. Note that second peaks in the plasma concentrations of aglycones were observed. This could be due to delayed absorption of aglycones hydrolyzed from glycosides by microflora in the gastrointestinal tract and enterohepatic circulation of anthraquinones [16]. A number of published studies have reported pharmacokinetics of anthraquinones. However, we found that most of these studies focused on determining aglycones. Pharmacokinetics of anthraquinone glycosides is rarely available. Wang et al. [12] recently reported the pharmacokinetics of anthraquinone aglycones and their glycosides in hyperlipidemic hamsters after administration of rhubarb. The plasma concentration-time profile patterns of anthraquinones were similar to our study even though the composition (dose ratio of compounds) of rhubarb extract might be quite different from *R. acetosa* extract and physiological differences between rats and hamsters probably exist. T_max_ values of glycosides were slightly longer than aglycones. Similar to our results, emodin glucoside was not detected even though emodin could be detected until 36 h. It is suggested that emodin glycoside probably rapidly hydrolyzed to emodin and was poorly absorbed as an intact form in the gastrointestinal tract. 

Our pharmacokinetic study has some limitations. The number of animals (*n* = 3) is not enough to achieve statistically significant pharmacokinetic parameters after administration of an herbal product. The pharmacokinetic parameters obtained in this study might b not sufficient to represent the animal population. Nevertheless, this study showed the possibility that our bioanalytical method could be used in pharmacokinetic studies of *R. acetosa* extract. Another issue to consider is that *R. acetosa* extract contained a number of other compounds besides anthraquinones. Further studies with a large sample size and studies of the effects of other compounds on the pharmacokinetics of anthraquinones are needed for better understanding of the pharmacokinetics of anthraquinones.

## 5. Conclusions

A simple and sensitive LC-MS/MS method for the determination of the glycosides and aglycones of anthraquinones in rat plasma was developed. The method was acceptably validated and applied to a pharmacokinetic study of anthraquinones after oral administration of *R. acetosa* extract in rats. The absorption of the glycosides of anthraquinones in an intact form was confirmed in the pharmacokinetic study. The results of this study could be relevant to a better understanding of the pharmacokinetics and pharmacodynamics of anthraquinone glycosides and aglycones.

## Figures and Tables

**Table 1:** Summary of the MS/MS parameters.

Compounds	MRM Transition (*m*/*z*) ^a^ Precursor Ion → Product Ion	Fragmentor (V)	Collision Energy (V)
E	269 → 225	145	20
EG	431 → 269	150	24
C	253 → 225	175	22
CG	415 → 253	89	13
P	283 → 240	157	16
PG	445 → 283	95	5
IS (Diclofenac)	294 → 250	65	1

^a^ MRM transitions refer to the reference [12]. MRM, multiple reaction monitoring; E, emodin; EG, emodin-8-*O*-β-d-glucoside; C, chrysophanol; CG, chrysophanol-8-*O*-β-d-glucoside; P, physcion; PG, physcion-8-*O*-β-d-glucoside; IS, internal standard.

**Table 2:** Accuracy and precision of anthraquinones in rat plasma (*n* = 6). RSD: relative standard deviation.

Analyte	Nominal Concentration (ng/mL)	Intra-Batch			Inter-Batch		
		Mean Calculated Concentration (ng/mL)	Accuracy (%)	RSD (%)	Mean Calculated Concentration (ng/mL)	Accuracy (%)	RSD (%)
P	20	20.5	102.5	8.83	21.3	106.4	11.2
	60	58.0	96.7	4.69	63.4	105.6	7.72
	150	156	104.2	3.98	158	105.3	5.90
	300	308	102.6	2.80	312	104.1	5.54
E	1	1.10	110.1	13.5	1.05	104.6	11.1
	3	3.31	110.3	6.87	3.12	103.9	6.87
	150	155	103.4	2.32	154	102.8	2.66
	300	309	103.0	3.45	299	99.8	2.28
C	20	21.8	109.2	9.23	19.8	98.9	2.98
	60	64.1	106.9	7.79	59.3	98.9	5.75
	150	153	101.9	4.91	155	103.1	6.85
	300	303	101.1	3.94	321	107.0	4.20
PG	10	10.2	102.1	10.7	10.2	102.3	12.1
	30	32.1	107.1	6.83	31.7	105.7	5.72
	75	80.3	107.1	5.38	80.5	107.3	4.95
	150	160	106.5	4.13	159	106.2	4.89
EG	1	1.10	110.1	18.9	1.05	105.2	9.10
	3	3.35	111.8	5.49	3.27	108.9	12.9
	75	77.3	103.0	1.59	78.5	104.6	3.85
	150	156	104.1	1.24	153.9	102.6	4.34
CG	10	11.2	111.9	8.34	11.0	109.9	8.00
	30	31.5	105.0	4.13	31.6	105.4	5.79
	75	76.3	101.7	2.72	76.5	102.0	3.16
	150	154	102.9	3.87	157	104.5	2.12

**Table 3:** Extraction recovery and matrix effect of anthraquinones in rat plasma (*n* = 3).

Analyte	Nominal Concentration (ng/mL)	Extraction Recovery (%)		Matrix Effect (%)	
		Mean	RSD	Mean	RSD
P	60	106.5	4.85	185.6	2.29
	150	106.4	1.23	185.1	3.13
	300	98.6	1.49	183.9	3.92
E	3	101.3	2.74	70.1	6.65
	150	105.6	0.82	87.0	1.46
	300	100.4	1.90	92.3	2.77
C	60	112.7	8.71	143.0	1.04
	150	102.1	1.07	144.3	1.62
	300	96.6	1.04	144.6	4.11
PG	30	106.4	13.0	42.5	8.41
	75	107.4	5.60	44.3	6.07
	150	96.6	3.14	51.4	2.33
EG	3	97.6	3.48	286.5	4.29
	75	100.5	0.77	277.6	3.60
	150	97.6	1.25	277.8	2.84
CG	30	96.0	1.36	113.8	6.64
	75	96.6	6.01	121.9	3.24
	150	96.5	6.89	126.7	2.02

**Table 4:** Stability of anthraquinones in rat plasma (*n* = 3).

Analyte	Conc. (ng/mL)	Short Term Stability (%)		Long Term Stability (%)		Freeze and Thaw Stability (%)		Processed Sample Stability (%)	
		Accuracy	RSD	Accuracy	RSD	Accuracy	RSD	Accuracy	RSD
P	60	104.9	2.05	107.3	5.31	103.6	8.18	102.2	9.10
	150	98.9	5.64	100.1	2.34	103.9	4.48	101.6	2.87
	300	103.8	2.39	98.0	2.00	104.2	3.21	102.7	5.76
E	3	103.2	3.46	106.8	4.25	107.5	2.38	108.2	5.26
	150	100.7	2.55	107.8	1.87	104.5	4.32	108.0	5.96
	300	101.8	2.44	107.7	0.93	102.0	3.47	104.6	3.60
C	60	103.5	6.23	104.3	8.67	89.7	0.76	101.5	9.60
	150	98.6	1.07	97.9	7.67	91.8	3.94	103.7	5.43
	300	101.2	2.97	102.6	2.98	92.4	8.25	102.3	6.47
PG	30	98.9	9.48	97.7	5.64	101.5	9.06	98.8	6.33
	75	91.8	6.60	98.5	5.36	94.3	6.57	92.7	4.64
	150	96.7	1.62	97.1	2.85	93.6	2.16	96.8	1.19
EG	3	99.8	5.11	102.9	4.02	100.4	6.39	91.5	3.11
	75	108.5	2.79	107.9	1.54	112.3	3.60	107.9	2.88
	150	98.9	1.81	102.8	2.14	100.9	1.69	100.3	4.14
CG	30	97.6	9.29	97.5	4.04	98.3	4.81	91.1	2.45
	75	96.9	0.56	100.6	1.38	101.0	2.23	100.2	7.38
	150	101.8	5.27	101.5	2.52	97.0	5.66	96.7	5.93

**Table 5:** The pharmacokinetic parameters of anthraquinones after oral administration of *R. acetosa* extract to rats at a dose of 2 g/kg (*n* = 3).

Analyte	AUC_0–last_^a^ (ng h/mL)	C_max_ (ng/mL)	T_max_^b^ (h)	MRT (h)	t_1/2_ (h)
C	265.6 ± 70.9	155.6 ± 86.0	0.25 (0.25–0.5)	2.4 ± 0.2	3.9 ± 0.6
E	1165 ± 336.1	123.5 ± 41.7	0.25 (0.25–0.75)	7.7 ± 3.0	NA
CG	158.0 ± 12.3	28.7 ± 4.7	2 (0.75–2)	2.4 ± 0.08	4.8 ± 0.5
PG	82.8 ± 13.8	20.5 ± 1.4	0.75 (0.5–2)	2.7 ± 0.6	6.2 ± 3.9

The values were expressed as mean ± standard deviation except T_max_. ^a^ The last measured time points for C, E, CG, and PG were 6, 24, 8, and 6 h. ^b^ Median (range). AUC_0–last_, total area under the plasma concentration–time curve from time zero to last measured time; C_max_, maximum plasma concentration; T_max_, time to reach C_max_; MRT, mean residence time; t_1/2_, half-life; NA, not available.

**Table 6:** Comparison with reported analytical methods for aglycones and glycosides of anthraquinone.

Analytical Condition		Our Method	Lin et al. [8]	Wang et al. [12]	Ma et al. [15]
Sample volume		20 μL	25 μL	25 μL	100 μL
Sample preparation		Protein precipitation	Solid phase extraction	Liquid-liquid extraction	Liquid-liquid extraction
Target compounds		E, C, P, EG, CG, PG	EG, E	E, C, P, EG, CG, PG	EG, E
LLOQ (ng/mL)	E	1	1	2	9.6
	C	20	-	50	-
	P	20	-	50	-
	EG	1	1	2	33.7
	CG	10	-	2	-
	PG	10	-	1	-

## Abbreviations

LC-MS/MS	liquid chromatography-tandem mass spectrometry
MRM	multiple reaction monitoring
E	emodin
EG	emodin-8-*O*-β-d-glucoside
C	chrysophanol
CG	chrysophanol-8-*O*-β-d-glucoside
P	physcion
PG	physcion-8-*O*-β-d-glucoside
